# The Hidden Economic Consequences of Migraine to the UK Government: Burden-of-Disease Analysis Using a Fiscal Framework

**DOI:** 10.36469/001c.87790

**Published:** 2023-10-03

**Authors:** Rui Martins, Samuel Large, Rachel Russell, Gary Surmay, Mark P. Connolly

**Affiliations:** 1 Health Economics Global Market Access Solutions LLC, St.-Prex, Switzerland; 2 University Medical Center Groningen, Groningen, Netherlands; 3 Pfizer Ltd, Tadworth, UK; 4 Pfizer Biopharmaceuticals Group, New York, New York, USA; 5 Health Economics Global Market Access Solutions Earl, St-Prex, Switzerland; 6 University Medical Center Groningen, University of Groningen, Groningen, The Netherlands

**Keywords:** migraine, burden of disease, fiscal analysis, economic evaluation, headache, work activity

## Abstract

**Background:** Migraine is a highly prevalent and incapacitating neurological disorder associated with the highest global disability burden in people aged 15 to 49 years. Europe has the fourth-highest prevalence of migraine, after North America, South America, and Central America, and above Asia and Africa. Migraine leads to relatively modest direct healthcare expenditure but has substantial indirect costs due to reduced productivity.

**Methods:** The economic burden of migraine was estimated in comparison with the general population of the United Kingdom (UK) using an analytical fiscal modeling framework applying the government cost perspective. Published measures of migraine’s impact on labor participation were applied to rates of economic activity/inactivity of the general population. The model estimates lifetime changes to earnings from employment, direct and indirect taxes paid, and financial support requirements over the life course. Incremental differences between those affected and unaffected by migraine are reported as net fiscal consequences to public accounts. Fiscal costs are reported as the discounted average per capita over a 20-year time horizon and for the entire annual UK cohort with prevalent migraine.

**Results:** People affected by migraine are more likely to be absent from work, unemployed, and disabled, and to retire early. A 44-year-old individual affected by migraine was associated with £19 823 in excess fiscal costs to the UK government, £1379 per year living with the condition, compared with someone not affected by the disease. Annually, migraine was estimated to represent £12.20 billion to the public economy, approximately £130.63 per migraine episode. The model predicted annual productivity losses in the health and social care workforce to be £2.05 billion and total annual productivity losses to be over £5.81 billion.

**Conclusions:** This fiscal analysis monetizes the occupational consequences of migraine to the UK government, both in terms of lost tax revenue and transfer payments. The findings are substantial and useful to characterize disease severity and to inform the body of evidence considered by decision makers appraising the cost-effectiveness of health technologies.

## BACKGROUND

The bidirectional relationship between health and wealth are established constructs in the healthcare and economics literature. The effects of poor health due to acute and chronic diseases can manifest in many ways that diminish lifetime earnings and increase dependency on public benefits support.^1^ The consequences of acute health events such as stroke and myocardial infarction can easily be measured and are often well documented.^2^ Health conditions with more subjective manifestations, particularly chronic conditions such as migraine, are commonly underdiagnosed and undertreated, reducing an individual’s ability to remain active in the workforce.^3^

Migraine is a neurological condition that commonly appears early in life but shapes the choices people make throughout their lifetime, translating to quantifiable economic losses. Unlike definitions of migraine used in the United States,^4^ neither the World Health Organization,^5^ European Union,^6^ nor the United Kingdom (UK)^7^ acknowledge key comorbidities associated with migraine, including regular occurrence of mental health disorders and an increased risk of cardiovascular disease. Therefore, the societal and economic impact of migraine may be underestimated by policymakers.

Previous studies have shown that migraine can influence educational attainment and school grades, suggesting a human capital impact that endures into adulthood.^8^ Migraine symptoms are highly disabling, disrupting normal working patterns and causing productivity losses.^9^ This in turn influences career progression, with individuals affected by migraine reporting feeling overlooked for promotions or simply not applying because of interruptions in their work patterns.^10,11^

The categorization of migraine severity has progressed over time^12^ but often^11^ considers the frequency of monthly migraine days (MMDs) or monthly headache days (MHDs) experienced by individuals. According to the most recent definition, episodic migraine (EM) is present in people with fewer than 15 MHDs over the past 3 months, with some being migraine.^12^ Chronic migraine (CM) has been defined as the occurrence of 15 or more MHDs over 3 months, 8 of which are migraine episodes per month.^13^ The current threshold of 15 MHDs may nonetheless provide insufficient detail to distinguish between CM and EM, with evidence suggesting a similar burden in people having 8 to 14 and 15 to 23 MHDs.^14^ In addition, shifting between CM and EM is thought to be common.^15^ Understanding migraine severity is crucial to accurately estimate clinical and occupational outcomes, all of which impact the perceived economic burden of the disease.

Based on a 2003 survey conducted among the population aged 16 to 65 years in mainland England, the 1-year prevalence of migraine with or without aura was 14.3% among the adult population.^16^ Worldwide, the annual prevalence of migraine was estimated to be 15%,^9^ with CM representing 5% to 6% of all cases.^17,18^ Migraine is a primary headache disorder predominantly impacting individuals in their productive years, with 3 in every 4 affected being female.^13,19^ Migraine attacks in women tend to be more frequent than those in men, and the attacks are more severe, have a longer duration, and are more challenging to treat.^20^ Migraine prevalence appears to increase until 40 years of age and then decline in older adulthood, particularly after menopause in female patients.^20,21^

Migraine symptoms include pain, visual disturbances, photophobia, and sickness,^22^ which deeply impact one’s personal, social, and professional functioning, and can lead to isolation.^23^ A growing body of evidence identifies comorbidities associated with migraine, including the regular occurrence of mental health disorders, increased risk of cardiovascular disease, and sleep disorders.^24^ However, the fact that these comorbidities are not consistently acknowledged as associated with migraine affects clinical care^25^ and risks both the societal and economic impact of migraine being underestimated by policymakers.

Due to its highly incapacitating symptoms and high prevalence, migraine is thought to be responsible for 5.6% of the total number of years lived with disability worldwide, surpassed only by low back pain among level IV disabling conditions.^26^ In the UK, headaches and migraines are the origin of 5% of all UK sickness absences,^27^ and the average migraine-associated disability-adjusted life-year burden is thought to be 10% higher than the average of countries with similar economic profiles.^11^

The burden of migraine on the labor market is often reported in terms of absenteeism and presenteeism metrics, monetized using a human capital approach.^23,28,29^ Although informative, this approach captures only short-term work disruptions and does not consider permanent employment transitions that people might make due to migraine and occupational disability related to the condition; therefore, it does not capture the full economic burden caused by migraine episodes. In light of financial pressure induced by increasing healthcare costs, aging populations, and shrinking numbers of working-age adults, the issue of health and social systems sustainability is of great concern, particularly in countries such as the UK, where health funding is mostly funded through taxation. The goal of this study is to utilize existing evidence of the effect of migraine on labor participation and an established fiscal framework^30^ to estimate the economic consequences of migraine to the UK government (ie, fiscal burden).

## METHODS

### General Considerations

**Figure 1.**
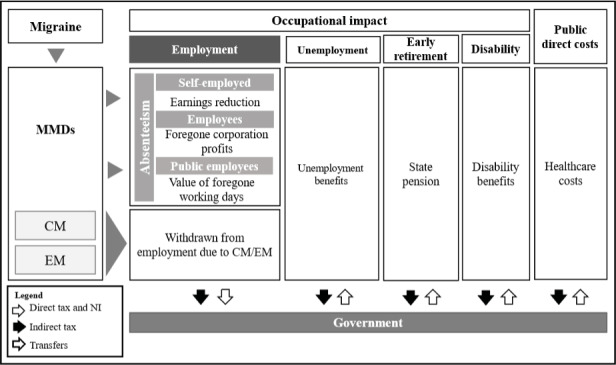
Model Diagram Abbreviations: CM, chronic migraine; EM, episodic migraine; MMDs, monthly migraine days; NI, National Insurance.

A modeling framework developed in Microsoft Excel was used to simulate and compare the fiscal pathways of people living with and without migraine. The analysis focused on fiscal consequences to capture the economic impact of the disease on the monetary exchanges between the government and individuals, consisting essentially of tax revenue and transfer payments (disability benefits, pensions, and public healthcare expenditure) (**Figure 1**). Model results did not include direct economic losses to the private sector but accounted for foregone government tax income due to migraine-related productivity losses in the private sector. A general description to the modeling approach is reported in the following sections. More intricate details, including most model inputs, are included in the **Supplemental Materials**. This economic analysis is reported in agreement with the Consolidated Health Economic Evaluation Reporting Standards (CHEERS) statement.^31^

### Modeling Framework

The modeling exercise comprised 2 parts, with the first estimating the longitudinal fiscal consequences of migraine in a cohort aged 44 years, with 84.7% female,^17^ over a 20-year time horizon (longitudinal model). The second part estimated the annual fiscal consequences of migraine in the entire UK population, using the current demographic distribution and published migraine prevalence metrics (population model). In both the longitudinal and population models, people affected by migraine were compared with demographically identical cohorts unaffected by the condition. In the longitudinal model, mortality was implemented using a life table method informed by annual age- and gender-specific probabilities of death.^32^ It was assumed that migraine was associated with no excess mortality.^33^ In the population model, the number of annually affected individuals was calculated by multiplying age- and gender-specific migraine prevalence figures^11^ by matching age and gender UK head counts for 2022.^34^

**Chronic and episodic migraine.** The model used a 5.3% to 94.7% distribution of EM to CM, respectively, sourced from the UK cohort of the International Burden of Migraine Study.^17^ A similar ratio of CM to EM (CM 5.5%/5.7%) was found by large international studies.^18,35^ Although the model uses age-specific inputs of migraine prevalence, it was assumed that the distribution of CM to EM is constant in relation to age. Relative measures of the effect of CM or EM on occupational outcomes described below were extracted using author-defined classifications of migraine severity and applied to the cohort with matching severity.

**Labor market participation.** In both models, the likelihood of occupational outcomes was obtained by applying age- and gender-specific annual rates of labor participation^36^ to the number of individuals in that age band. Economically active individuals could be employed or unemployed. Those employed could be employees (private or public sectors) or self-employed based on UK employment figures.^36^ The breakdown of economic inactivity (due to disability or early retirement) in the UK general population was obtained from public sources.^37,38^ Retirement age was assumed to start at 66 years for individuals no longer remaining active.^36,39^

**Absenteeism losses to the UK government.** Absenteeism-related losses are an important component of the economic burden of migraine.^11,40^ Commonly, burden-of-migraine analyses monetize absenteeism by assigning average hourly wages to lost productivity time,^11,40^ not distinguishing public from private sector losses. When applying a government perspective of costs, this distinction is important. Different approaches were therefore employed to estimate the economic consequences of absenteeism in public sector employees, private employees, and self-employed workers. In the UK, public and private employees still receive their monthly wages despite short-term episodes of sickness and associated foregone labor. These expenses are usually borne by the employee. When the employee was in the public sector, these costs were considered a monetary loss to the government, estimated from a human capital approach. This was applied to public sector employees including health and social care staff not providing direct care. Staff providing direct health and social care require replacement to ensure service continuity, often at higher hourly costs compared with substantive staff. Replacement costs were calculated by applying absenteeism rates to national wages increased by 55.0% to reflect agency fares.^41^ Because labor is provided by the replacement staff, only replacement costs were added to the totals, with substantive staff’s salary related to the absenteeism period being excluded to avoid double counting. Replacement staff earnings were subject to the same rate of direct and indirect taxation, which are discussed in the fiscal consequences section below.

In private sector employees, absenteeism costs, derived through a human capital methodology, were seen as a reduction in corporate profits. Foregone corporate taxes were considered a financial loss to the government.

In self-employed workers, absenteeism costs were calculated using a human capital approach. Foregone taxation due to reduced earnings was added as a fiscal loss. The inputs informing absenteeism calculations are included in the **Supplemental Material**.

The fiscal losses associated to health and social care workforce absenteeism represent direct costs to the National Health System and were therefore disaggregated from the results.^42^

**Effect of migraine on labor participation.** Measures of the effect of migraine on occupational outcomes were sourced from published studies identified through a targeted literature search conducted on PubMed and supplemented by Google Scholar searches and by cross-checking references from systematic reviews and key burden of migraine publications. Data specific to the UK were identified for migraine-related absenteeism only. Evidence of the effect of migraine on reduced employment, increased disability, and early retirement was obtained from publications from other developed countries. We assumed that the relative effect of migraine on occupational outcomes would be similar to that of developed countries with similar economic profiles. Inputs selected for use in the model are shown in **Table 1**. The likelihood of occupational outcomes in the cohort affected by migraine were calculated by applying the inputs depicted in **Table 1** to the general population rates of employment, absenteeism, disability, and early retirement. More details about the targeted literature search, including the search strategy, and implementation of the selected inputs in the model were included in the **Supplemental Materials**.

Table 1. Measures of the Relative Effect of Migraine on Occupational Outcomes

**Table 1.** Measures of the Relative Effect of Migraine on Occupational Outcomes

**Outcome**	**Migraine Severity**	**Mean**	**SE**	**Source**
Current employment (full-⁠ or part-⁠time)	Low EM (0-2 MHDs) [Reference]			Stewart et al (2010)
	Moderate EM (3-9 MHDs) [RR]	0.940	0.027	
	High EM (10-14 MHDs) [RR]	0.86	0.062	
	CM (>14 MHDs) [RR]	0.810	0.039	
	EM (10-14 MHDs) [RR]	0.993	–	Calculated^a^
Full-time/part-time ratio	EM (≤ 14 MHDs)	52.3%	0.5%	Buse et al (2010)
	CM (>14 MHDs)	37.8%	1.9%	
	CM vs EM [RR]	0.723	--	Calculated^b^
Absenteeism (% work time missed)	Controls (no migraine)	1.9%	10%^c^	See note^d^
	EM (4-7 MHDs)	8.0%		Vo (2018)^43^
	EM (8-14 MHDs)	22.2%		
	CM (>14 MHDs)	19.7%		
	EM (≤ 14 MHDs)	12.6%	–	Calculated^e^
Disability	EM (≤ 14 MHDs)	11.1%	0.3%	Buse et al (2010)
	CM (>14 MHDs)	20.0%	1.6%	
	CM vs EM [RR]	1.802	--	Calculated^f^
Early retirement	HFEM (≥8 MHDs but <15 MHDs)	20.8%	3.2%	Chalmer (2020)^44^
	CM (≥8 MHDs but ≥15 MHDs)	33.5%	3.8%	
	CM vs EM [RR]	1.611	--	Calculated^g^

^a^It was assumed that having fewer than 10 MHDs would not affect one’s probability of staying in employment. Employment reductions was therefore applied only in people affected by EM having at least 10 MHDs and in those with CM. The proportion of EM with at least 10 MHDs (296/6267; 4.7%) was informed by Steward et al.^45^

^b^Ratio between proportion of people with CM in full-time employment and EM in full-time employment (0.378/0.523).

^c^Assumed to be 10% of the mean.

Abbreviations: CM, chronic migraine; EM, episodic migraine; HFEM, high-frequency episodic migraine; MHDs, monthly headache days; RR, relative risk, SE, standard error.

### Fiscal Consequences

**Earnings**. The monetary consequences associated to each occupational state (wages, government benefits, taxes) were ultimately assigned to employed, unemployed, and disabled or retired individuals. Gross earnings from employment were stratified according to part-time or full-time status, age, and gender distribution.^46^ In the base case, self-employed individuals were deemed to have similar earnings to employees of the same age and gender. In a scenario analysis, we have accounted for evidence suggesting that full-time self-employed males, part-time self-employed males, full-time self-employed females, and part-time self-employed females can earn 31.9%, 11.2%, 31.4%, and 46.4% less than their employee counterparts, respectively.^47^

**Transfers from government.** The excess of individuals out of employment due to migraine were assigned a weekly job seeker’s allowance (£61.05 if ≤24 years of age; £77 otherwise).^48^ Disabled individuals in the population affected and unaffected by migraine were assigned the weekly value of personal independence payment from all disability causes. The proportion of individuals with migraine-related excess disability were assigned the weekly value of personal independence payment.^49^ In face of identical mortality in those affected and unaffected by migraine, and in the absence of evidence suggesting that the economic value of old-age pensions would be differential, we have not included these in the model. Individuals retiring early were assumed to receive state pension transfers.^50^

**Taxes**. Direct taxes were calculated as a proportion of total earnings using the UK tax wedge of 30.4%.^51^ The tax wedge includes all the expenses associated with one job including National Insurance contributions by the employee and employer and employment taxes paid by the employee. Indirect taxes resulting from everyday consumption were applied as a 12.4% proportion to all income from earnings and transfers.^52^ Corporation taxes were applied at a 19.0% rate to foregone profits due to absenteeism in the private sector.^53^

**Healthcare costs.** Healthcare expenditure was considered one of government expenses benefiting individuals. The average cost of managing one or more episodes of migraine was sourced from the National Institute for Health and Care Excellence (NICE) single-technology appraisal for erenumab.^54^ Unit costs for resources required in the calculations were updated to most recent values. The frequencies of MMDs in people with EM and CM were sourced from published distributions.^55^ At the time of writing, there is no source to inform the proportion of the UK migraine population seeking medical help.^11^ Evidence from the US and Germany suggesting that only approximately 46% of individuals seek medical health^56–58^ was used to determine the proportion of the population incurring healthcare costs. It is likely that on a migraine day, most patients will use medication in an attempt to suppress symptoms.^56^ The cost of medication in the 54% not having a prescription was assumed to be obtained over-the-counter, representing an out-of-pocket expense not incurred by the government.

**Model results.** Results were reported as Incremental Fiscal Consequences (IFC) calculated as the difference in net present values between people affected and unaffected by migraine^30^ (**Equation 1**). The net present values of the fiscal consequences was calculated by summing the monetary value of tax revenue and total government transfers associated with the average fiscal pathway of individuals in each comparator (**Equation 2**). Transfers such as disability pensions and healthcare costs were represented as negative monetary values so to imply an expense to the UK government. Sources of revenue (from taxation) were represented as positive values (**Equation 3 and Equation 4**). The equations below synthesize the involved calculations.

(1)IFC=NPVMigraine−NPVNoMigraine

(2)NPVi=∑t0tTaxt−Transferst(1+r)t

(3)Taxt=Direct Taxt+Indirect Taxt+Healthcare Costs

(4)Transferst=Financial Supportt+Healthcare Costs

where i is migraine status, r is the 3.5% discount rate for costs and life-years,^42^ and t is time in years.

**Scenarios and sensitivity analyses.** We investigated the most influential inputs in the model by conducting one-way sensitivity analyses (OSA) on most inputs parameterizing the model. The OSA was implemented using Visual Basic for applications by varying the mean base case inputs by the upper and lower bounds of their 95% confidence intervals. The impact of the 10 most influential inputs was synthesized in a tornado diagram.

In addition, some of the model base case assumptions were also challenged in deterministic scenarios. To explore uncertainty around the income of self-employed individuals,^59^ data from the Office for National Statistics were used to reduce base case earning inputs.^47^ The fiscal impact of a hypothetical treatment reducing MMDs by 5% or 10% was investigated in separate scenarios. The fiscal burden of a cohort affected only by EM was also reported. Given the uncertainty around the proportion the migraine population using public healthcare resources, the base case value was varied to the arbitrary values of 20% and 70%.

## RESULTS

**Table 2** shows the incremental fiscal consequences and life-years referring to the longitudinal model. Results are shown per capita and were discounted at 3.5% annually after year 1. From the age of 44 and over a 20-year time horizon, the average excess fiscal burden to the UK government was estimated to be £19 834 per capita or £1379 per life-year of an individual with migraine, compared with someone not affected by the disease. Of these costs, 33.6% were due to foregone public sector productivity, 32.7% to foregone taxes, 29.9% to healthcare costs, and 3.8% were due to excess transfers from government. Over this 20-year period, each individual affected by at least 10 MMDs was predicted to be associated with £7434 in lost earnings from reduced employment.

**Table 2.** Base Case Results Cohort Model Comparing UK Migraine Population With General Population^a^

	**Migraine Population**	**General Population**	**Incremental**	**Fiscal Impact**
**Gross income from any employment**	£249 956	£257 391	-£7434	
Fiscal consequence				
Public sector absenteeism	-£7831	-£1173	-£6658	33.6% (loss)
Direct taxes from employment	£75 987	£78 247	-£2260	11.4% (loss)
Indirect taxes from employment	£30 995	£31 916	-£922	4.7% (loss)
Foregone corporation taxes	-£3895	-£583	-£3311	16.7% (loss)
Job seeker’s allowance	-£2035	-£1344	-£691	3.5% (loss)
Early retirement pension	-£1101	-£1066	-£35	0.2% (loss)
Disability pension	-£6336	-£6312	-£25	0.1% (loss)
Indirect tax from transfers	£922	£915	£7	0.0% (gain)
Healthcare costs	-£34 285	-£28 357	-£5929	29.9% (loss)
Total	£52 421	£72 244	-£19 823	
Life-years	14.371	14.371	0.00	
Incremental costs per life-⁠year with migraine			**-£1379**	

Negative values represent monetary losses and positive values sources of revenue to the UK government.

^a^All government costs and tax revenue were discounted at 3.5%.

**Figure 2.**
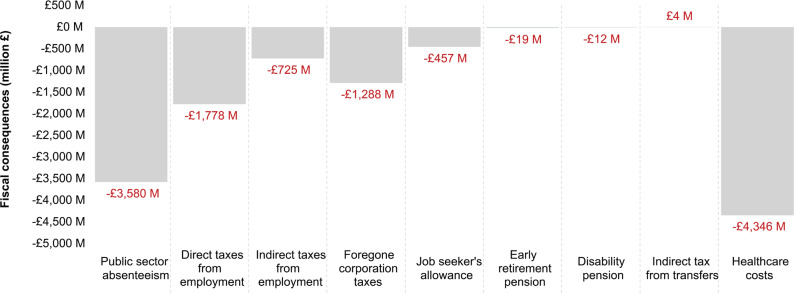
Base Case Results of the Population Model Comparing UK Migraine Population With General Population Discounted at 3.5%^a^ ^a^Negative monetary values represent a loss to the UK government. Indirect tax from transfers consist of consumption taxes resulting from government benefits received by individuals.

The results of the population model provide a cross-sectional picture of the overall fiscal burden to the UK government, associated to all individuals with prevalent migraine (**Figure 2**). A total of 10 535 224 UK residents aged 18 years and older were estimated to be affected by migraine annually. These individuals were predicted to experience 93 408 436 migraine days of all severities. We estimated that the cohort with prevalent migraine would be associated with £12 202 million in excess fiscal consequences in a single year, compared with an identical cohort unaffected by migraine. On average, each episode of migraine would represent a £130.63 loss to the public economy. Productivity losses related to absenteeism were expected to represent 47.6% (£5.8 billion) of the total fiscal burden. The public sector workforce accounted for 61.6% (£3.6 billion) of these losses, self-employed workers for 16.2% (£941 million), and employees in the private sector for 22.2% (£1.3 billion). Considering that health and social care workers represent 36.2% of the public sector workforce would imply £3.6 billion in migraine-related absenteeism losses annually due to direct care providers’ replacement costs (£727 million) and forgone labor (£2.9 billion).

Additional results, including the projected prevalence of migraine in the UK, the disaggregated fiscal consequences estimated by the population model, and the breakdown of absenteeism-related costs, are included in the **Supplemental Material**.

### One-Way Sensitivity Analysis

**Figure 3.**
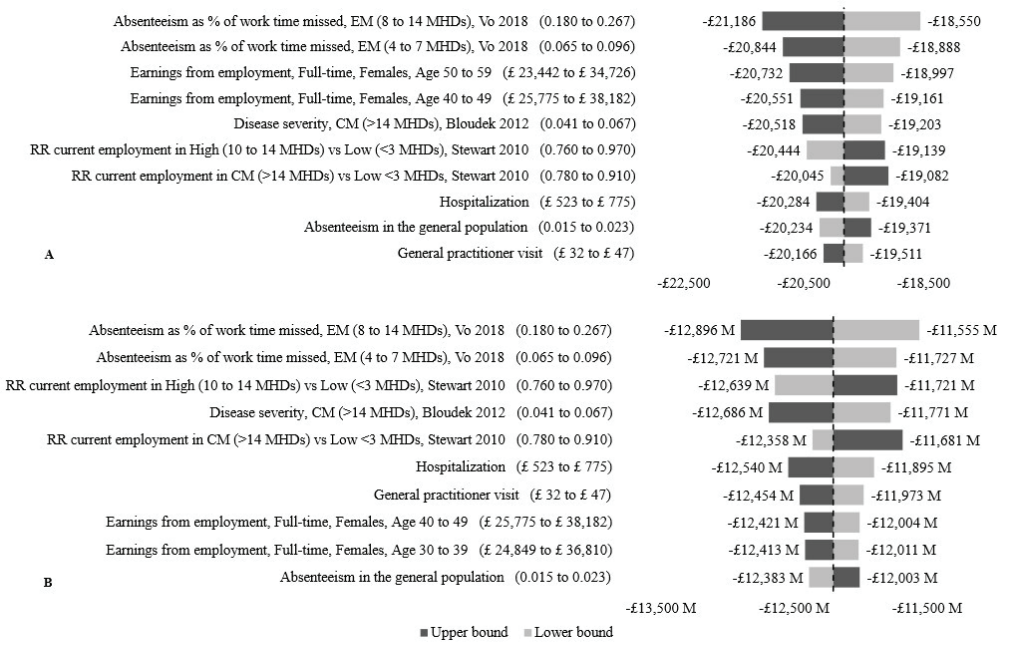
Tornado Diagrams of (**A**) Longitudinal and (**B**) Population Models Abbreviations: CM, chronic migraine; EM, episodic migraine; MHDs, monthly headache days, RR, relative risk.

The OSA conducted on the results of the longitudinal and population models are synthesized in the tornado diagrams shown in **Figure 3**. Model results were most sensitive to the absenteeism as percentage of work time missed in people with 4 to 7 and 8 to 14 MHDs. Varying these parameters increased or decreased the IFC by a maximum of 7% in the longitudinal and population models. Parameters affecting the relative risk of employment in people with 10 to 14 MHDs compared with less than 3 MHDs, and the value of employment earnings in females caused a less than 5% variation in IFC from base case. The remaining influential parameters varied the IFC by a maximum of 4% in both the longitudinal and population models.

### Scenario Analyses

Lowering the wages of self-employed workers would imply 3.2% and 3.1% lower absolute IFC in the longitudinal and population models, respectively. Reducing MMDs by 5% would generate 1.5% and 1.7% lower fiscal costs, which would represent savings of £291 per average person over the 20-year time horizon, or £209 million per year for the entire UK cohort. A 10% reduction in MMDs could lead to a 2.9% to 3.4% reduction in the incremental fiscal burden of migraine (ie, £583 per capita in the longitudinal model or £417 million annually) compared with individuals not affected by the disease. A hypothetical treatment, alleviating all CM cases to EM, would reduce IFC by 13%, or £2623 per person over the time horizon of the analysis or £1823 million per year, according to the population model. The incremental results from the longitudinal and population models associated to these deterministic scenarios are shown in **Table 3**. Varying the proportion of people using public healthcare resources to 20% led to an IFC 17% to 20% lower than baseline (£16 472 per person in the longitudinal model, or £9746 million for the entire population). Assuming 70% health resource utilization, this led to a 16% to 19% increase in fiscal burden, totaling £22 916 per person over 20 years, or £14 470 million per year for the entire migraine population.

**Table 3.** Incremental Results (in £) from Longitudinal and Population Models Associated With Deterministic Scenarios

**Scenario**	**Base Case**		**Lower Self- employed Wages**		**Therapy Reducing MMDs by 5%**		**Therapy Reducing MMDs by 10%**		**No CM**	
**Model**	**Long.**	**Pop.**	**Long.**	**Pop.**	**Long.**	**Pop.**	**Long.**	**Pop.**	**Long.**	**Pop.**
Public sector absenteeism	-6658	-3580 M	-6658	-3580 M	-6653	-3578 M	-6649	-3576 M	-6565	-3533 M
Direct taxes from employment	-2260	-1778 M	-1807	-1508 M	-2193	-1730 M	-2126	- 682 M	-909	-807 M
Indirect taxes from employment	-922	-725 M	-737	-615 M	-894	-706 M	-867	-686 M	-371	-329 M
Foregone corporation taxes	-3311	-1288 M	-3311	-1288 M	-3309	-1288 M	-3307	-1287 M	-3266	-1271 M
Job seeker’s allowance	-691	-457 M	-691	-457 M	-670	-443 M	-650	-430 M	-279	-184 M
Early retirement pension	-35	-19 M	-35	-19 M	-33	-18 M	-31	-17 M	0	0 M
Disability pension	-25	-12 M	-25	-12 M	-25	-12 M	-25	-11 M	-24	-11 M
Indirect tax from transfers	7	4 M	7	4 M	7	4 M	7	4 M	3	1 M
Healthcare costs	-5929	-4346 M	-5929	-4346 M	-5761	-4223 M	-5593	-4100 M	-5789	-4244 M
Total	-19 823	-12 202 M	-19 185	-11 822 M	-19 531	-11 994 M	-19 240	-11 785 M	-17 200	-10 379 M
Changes from base case	-	-	3.2%	3.1%	1.5%	1.7%	2.9%	3.4%	13%	15%

Abbreviations: CM, chronic migraine; Long., longitudinal; M, million; MMDs, monthly migraine days; Pop., population.

## DISCUSSION

The UK takes a broad societal approach to assessing most investments through the HM Treasury *Green Book* except for medicines, with only a narrow definition of the value they create considered by the health technology assessment process. Despite the wider socioeconomic burden migraine presents, at both a pan-EU and national level, migraine is relatively absent from health policy discussions. The analysis described here illustrates how government public accounts can be impacted by migraine sufferers. While many analyses focus on health costs, we have extended the analysis to consider a broader range of costs that fall onto government, namely, lost tax revenue from reduced employment and increased spending on public benefits programs for those unable to work due to migraine. As described in this study, the fiscal burden of migraine to the UK government for an average 44-year-old results in £19 823 in fiscal losses over a 20- year time horizon, or £1379 per life-year with migraine. A substantial share of the burden was predicted to relate directly to healthcare costs (29.9%-35.6%); nonetheless, 64.4% to 70.1% of the fiscal burden relates to productivity losses and consequential forgone taxation. This is in line with previously published studies identifying decreased productivity as the largest cost component of the economic burden of migraine.^60–62^ While our analysis illustrates the impact within a single cohort, these costs are likely an underestimate, as the impact of many health conditions can lead to consequences spreading across economic sectors and through several generations.^45,63^ An example is the impact of migraine felt among the NHS’s own staff. In a single month in 2021, 2.3% of total NHS staff absences were due to migraine or headache; the migraine absence accounted for full-time equivalent of 51 179 days lost.^64^ It is also important to highlight that a substantial share of the burden of migraine falls on women, which may further amplify existing gender inequalities. This can be particularly important after menopause, which is known to exaggerate migraine symptoms, and can contribute to increased sickness absence, reduced productivity, and early job discontinuation of labor activities.^65^

Migraine is a condition with high prevalence and important consequences to individuals’ well-being. It disrupts the ability to perform daily activities, impacting normal occupational pathways and ultimately affecting the economic relationships between individuals and the government. Reduced labor participation leading to reduced tax contributions can increase financial hardship and the need for financial support.^14,66,67^ In our analysis, we have attempted to capture the fiscal burden of migraine in the UK; however, these findings are not thought to be unique to the UK, and we would expect similar fiscal consequences to be observed in countries with similar migraine prevalence and social support systems.^26^ In countries with more fragile economies and welfare systems, migraine can be underdiagnosed and undertreated, aggravating the burden of disease.^26,68^ Considering these important relationships, governments may fiscally benefit from improved health status in this population and reducing disability costs that are known to exist in this population.^69^

Conducting public economic assessments of health conditions reveals how all members of society are linked through public economic accounts. In advanced economies, people who are unable to work due to poor health are dependent on the productive output of those members of society who remain working to fund tax-financed public programs. Consequently, when one person withdraws from the work force due to migraine or any other health matter, the remaining work force must contribute more to public systems to continue funding government programs. For conditions with large epidemiologic footprints, this can create considerable fiscal drag for governments that must be funded by the remaining workers in the economy. Each worker who is unable to fulfill their fiscal lifetime of taxes paid can contribute to higher taxes for other members of the economy, which can increase the deadweight loss to the economy. Therefore, any intervention that prevents people with migraine withdrawing from work can create fiscal space for government and offer a range of economic benefits for society. In an era of aging populations and the need to retain the productive output of the remaining workers, these issues should be considered priority policy interventions for governments.^70^

Migraine is thought to impact many aspects of employment for sufferers across economic sectors. However, an accepted limitation of our analysis is that it does not fully consider the burden of absenteeism to the private sector, as we only included absenteeism-related foregone taxation. In the private sector, the costs of absenteeism are mostly absorbed by employers experiencing reduced revenue due to increased administrative costs and productivity reductions. In general, employees will retain their earnings during migraine-related short-term work absences. Consequently, this does not directly affect the government and hence does not require quantifying within a government perspective framework as we have described. Based on previously published studies taking a societal perspective, similar absenteeism losses should be expected in public and private sectors employees.^11,60^

Comparison of our results with those from other burden-of-disease studies must be done with caution due to the unique governmental perspective of costs we have used. This fiscal analysis estimated migraine to afflict approximately 10.5 million UK individuals, which is comparable to results from other studies.^11^ Also, this analysis predicted that employment and productivity-related fiscal costs would add to approximately £5.8 billion annually. Despite being composed of different elements, this value is in line with the £5.6 to £8.8 billion estimated productivity losses reported by the Work Foundation. Finally, we have used direct healthcare unit costs included in several single technology appraisal submissions to NICE and have derived countrywide annual direct healthcare costs of £4.3 billion. This value makes intuitive sense given the abovementioned prevalence but is substantially different from that reported in other studies.^11^ The input leading to such disparity is, of course, the proportion of the migraine population reaching out to the healthcare services. To the best of our knowledge, the proportion of patients remaining untreated is not known. We have varied this input in deterministic scenarios, but the resulting output should also be interpreted with caution. An increase in healthcare utilization is likely to lead to higher costs of health care but would also lead to positive health effects, which were not accounted for.

Our analysis has notable strengths and limitations. We used an established framework^45,63^ to demonstrate how migraine impacts the public economy, and our methods were transparently reported and replicable. A public UK data set and evidence from peer-reviewed publications were utilized to ensure the face validity of our results. We have used deterministic scenarios to investigate parameter uncertainty and the value of hypothetical therapies reducing the burden of migraine, which we believe is a useful format for decision makers. The study is limited by the fact that some important inputs are not specific to the UK, namely, the relative effect of migraine on the likelihood of labor participation and discontinuation. The impact of varying these inputs was subjected to scrutiny using sensitivity analyses, which were also reported. It is likely that for people living with migraine, labor force participation can be influenced by the job market and accessibility to state benefits when ill. As these factors are often context- and country-specific, the results may vary when applying this approach in other countries. Nonetheless, it seems reasonable to assume that diseases will have a similar effect on occupational outcomes across countries with similar economic profiles.

## CONCLUSION

This analysis has estimated the excess costs to the UK government resulting from changes to labor participation due to migraine symptoms. The fiscal burden was mostly due to productivity losses and foregone taxation. Personal losses from foregone employment earnings were also substantial. Public expenditure in migraine-related healthcare expenditure represented approximately 5% of total governments costs, yet there is no mention of migraine as a priority in the Public Health England 2020-2025 strategy.^71^ Our results allow policy makers to perceive the impact of a disease such as migraine on other sectors of the economy and should be considered to inform decisions about investments in health technologies. In face of the important consequences of diseases such as migraine on the labor market, and the challenges of aging populations, we suggest that future research focus on assessing how disease impacts occupational outcomes as these provide important information about how severely conditions impact individuals’ lives.

### Author Contributions

R.M. and M.P.C. were involved in the conception and design of the study, data acquisition, interpretation of the results, manuscript drafting and critical revision. R.M. was responsible for developing the economic model. S.L., R.R., and G.S. were involved in the conception and design of the study, interpretation of the results, and critical revision of the manuscript. S.L. and R.R. also provided inputs to support the modeling.

### Disclosures

R.M. and M.C. are employees of Global Market Access Solutions and were paid consultants to Pfizer. The work reported will be included as supporting material for the doctorate of R.M. at the University of Groningen. S.L. and R.R. are employees of Pfizer and hold shares in the sponsoring firm. G.S. is an employee of Pfizer and reports no further conflicts. The authors retained full editorial control over the final submitted manuscript.

### Data Availability

The supporting materials for this modeling analysis have been cited in the main text and supplemental material. The literature search strategy used to inform the analysis has been provided in the supplemental material.

## Supplementary Material

Supplementary Online Materials This supplementary material has been provided by the authors to give readers additional information about
their work.
