# Supplementary material for: The Hidden Economic Consequences of Migraine to the UK Government: Burden-of-Disease Analysis Using a Fiscal Framework
Source: J Health Econ Outcomes Res. 2023 Oct 3;10(2):72–81. doi: 10.36469/001c.87790 (PMC10552723; doi:10.36469/001c.87790)
Supplement: Supplementary Online Materials — This supplementary material has been provided by the authors to give readers additional information about their work. [file jheor_2023_10_2_87790_182722.pdf]

The Hidden Economic Consequences of Migraine to the UK Government: Burden of Disease Analysis Using a Fiscal Framework

Supplemental Materials

Contents

1. UK Labour market participation 2
UK public sector as a share of the total workforce 3
1. Economic inactivity in the UK population 4
Early retirement 4
Disability 4
2. Targeted literature search 5
PubMed literature search strategy 5
3. Occupational burden of migraine and model implementation 6
Employment 6
Absenteeism 6
Early retirement 6
Disability 6
4. Fiscal consequences inputs 8
Earnings from employment 8
Absenteeism costs 8
Personal Independence Payment (PIP) 8
Pensions 9
Direct healthcare costs 9
5. Monthly migraine days 11
6. Additional results 12
7. References 14

1. UK Labour market participation

Table 1 depicts the age and gender-specific rates of labour participation in the UK general population.

Table 1 – Labour market status by age and gender

		All		Economically active		Employed		Employee		Self-employed	
	Age categories	Economically Active	Economi-cally inactive	Employed	Unemployed	Employee	Self-Em-ployed	Employee: Full-time	Employee: Part-time	Self-employed: Full-time	Self-employed: Part-time
**Males**	16 to 19	36.8%	63.2%	79.4%	20.6%	96.2%	3.8%	41.8%	58.2%	63.4%	36.6%
	20 to 24	75.1%	24.9%	88.6%	11.4%	92.8%	7.2%	78.9%	21.1%	82.2%	17.8%
	25 to 29	89.5%	10.5%	95.0%	5.0%	90.8%	9.2%	93.4%	6.6%	89.1%	10.9%
	30 to 34	93.3%	6.7%	97.1%	2.9%	87.7%	12.3%	94.9%	5.1%	91.5%	8.5%
	35 to 39	92.8%	7.2%	97.1%	2.9%	85.7%	14.3%	95.6%	4.4%	90.9%	9.1%
	40 to 44	92.2%	7.8%	97.2%	2.8%	83.8%	16.2%	94.1%	5.9%	90.1%	9.9%
	45 to 49	90.7%	9.3%	97.1%	2.9%	82.6%	17.4%	94.4%	5.6%	90.0%	10.0%
	50 to 54	88.4%	11.6%	96.9%	3.1%	80.8%	19.2%	94.0%	6.0%	87.0%	13.0%
	55 to 59	80.2%	19.8%	96.2%	3.8%	78.6%	21.4%	90.0%	10.0%	81.5%	18.5%
	60 to 64	62.6%	37.4%	95.4%	4.6%	74.6%	25.4%	80.1%	19.9%	70.5%	29.5%
	65 to 69	28.0%	72.0%	97.1%	2.9%	64.5%	35.5%	60.0%	40.0%	55.1%	44.9%
	70 to 74	12.3%	87.7%	98.8%	1.2%	54.1%	45.9%	37.1%	62.9%	34.6%	65.4%
	75 to 79	6.4%	93.6%	97.9%	2.1%	38.0%	62.0%	26.5%	73.5%	35.3%	64.7%
	80 and over	2.0%	98.0%	100.0%	0.0%	42.5%	57.5%	26.0%	74.0%	19.2%	80.8%
**Females**	16 to 19	39.4%	60.6%	79.2%	20.8%	99.0%	1.0%	25.3%	74.7%	0.0%	100.0%
	20 to 24	72.1%	27.9%	92.5%	7.5%	96.4%	3.6%	68.0%	32.0%	42.8%	57.2%
	25 to 29	84.1%	15.9%	96.2%	3.8%	95.0%	5.0%	80.1%	19.9%	59.8%	40.2%
	30 to 34	82.4%	17.6%	96.4%	3.6%	92.8%	7.2%	72.1%	27.9%	57.8%	42.2%
	35 to 39	83.4%	16.6%	96.7%	3.3%	89.9%	10.1%	64.7%	35.3%	43.3%	56.7%
	40 to 44	84.4%	15.6%	96.5%	3.5%	89.9%	10.1%	64.3%	35.7%	47.6%	52.4%
	45 to 49	82.8%	17.2%	97.0%	3.0%	88.1%	11.9%	66.2%	33.8%	55.0%	45.0%
	50 to 54	80.4%	19.6%	97.5%	2.5%	88.1%	11.9%	66.3%	33.7%	53.0%	47.0%
	55 to 59	72.3%	27.7%	96.5%	3.5%	87.4%	12.6%	59.9%	40.1%	49.7%	50.3%
	60 to 64	50.9%	49.1%	96.7%	3.3%	84.8%	15.2%	48.0%	52.0%	39.2%	60.8%
	65 to 69	20.6%	79.4%	98.3%	1.7%	78.4%	21.6%	31.2%	68.8%	25.5%	74.5%
	70 to 74	6.9%	93.1%	99.2%	0.8%	65.0%	35.0%	17.5%	82.5%	24.4%	75.6%
	75 to 79	2.9%	97.1%	98.6%	1.4%	56.5%	43.5%	11.9%	88.1%	16.1%	83.9%
	80 and over	0.8%	99.2%	100.0%	0.0%	45.6%	54.4%	11.5%	88.5%	24.2%	75.8%

Source: Annual Population Survey, ONS 2021 (ONS 2021)

UK public sector as a share of the total workforce

The total number of public sector employees by in industry is shown in Table 2. This total was combined with the age distribution of UK civil servants to estimate the head-count of public sector employees by age and gender (Table 3).

Table 2 – UK public sector employment by industry in 2021

	Industry type	Count	%
	Construction	34,000	0.6%
Public administration, defence, compulsory social security	HM Forces 3	159,000	2.8%
	Police (including civilians)	269,000	4.7%
	Public administration	1,121,000	19.8%
	Education	1,491,000	26.3%
Health and social work	National Health Service	1,846,000	32.5%
	Other health and social work	208,000	3.7%
	Other public sector	548,000	9.7%
	Total public sector	**5,674,000**	100.0%

Source: ONS 2021 (ONS 2021)

Table 3 – Estimation of public workers as a proportion of total UK employees

**Age categories**	**Civil service distribution of full time and part time [1]**				**Civil service distribution by age and gender [a]**		**Projected UK public workers count [b]**		**Total UK population of employees [c]**		**Public workers as a proportion of the UK population of employees [d]**	
	**Full-time**	**Part-time**	**Full-time**	**Part-time**								
	**Males**		**Females**		**Males**	**Females**	**Males**	**Females**	**Males**	**Females**	**Males**	**Females**
**16 to 19**	95.1%	4.9%	94.7%	5.3%	0.2%	0.2%	11,011	10,071	408,100	423,900	2.7%	2.4%
**20 to 29**	97.9%	2.1%	92.1%	7.9%	6.1%	6.6%	347,508	377,184	2,935,100	2,872,000	11.8%	13.1%
**30 to 39**	95.6%	4.4%	64.0%	36.0%	9.6%	11.8%	545,567	670,041	3,472,400	3,251,100	15.7%	20.6%
**40 to 49**	94.2%	5.8%	61.0%	39.0%	11.0%	14.0%	624,656	792,904	3,007,100	2,999,500	20.8%	26.4%
**50 to 59**	91.2%	8.8%	64.3%	35.7%	14.4%	17.4%	817,209	985,860	2,877,300	2,994,700	28.4%	32.9%
**60 to 64**	64.5%	35.5%	43.6%	56.4%	3.4%	3.5%	192,150	199,401	850,700	830,800	22.6%	24.0%
**65+**	46.3%	53.7%	30.1%	69.9%	1.0%	0.7%	58,813	41,626	420,500	373,100	14.0%	11.2%

[a] Calculated from the 2018 UK Civil Servants Annual Survey of 422,500 participants (ONS 2021)

[b] Calculated using the civil servants' distribution by age and gender (ONS 2018) and the total population of public sector employees (5,674,000) (ONS 2021).

[c] Sourced from the Annual Population Survey (ONS 2021)

[d] Calculated by dividing the projected UK public workers count by the UK population of employees' value (i.e., 11,011/408,100=2.7%)

1. Economic inactivity in the UK population

In the model, individuals with disease-related disability or early retirement and individuals retired due to old age were considered economically inactive.

Early retirement

The proportion of people retiring early in the general population was sourced from the Annual Population Survey (ONS 2021) (Table 4).

Table 4 – Early retirement

Age categories	Males			Females		
	**Total**	**Retired (n)**	**Retired (%)**	**Total**	**Retired (n)**	**Retired (%)**
**16 to 24**	1,424,800	0	0.0%	1,384,400	0	0.0%
**25 to 49**	889,500	3,400	0.4%	1,789,500	6,300	0.4%
**50 to 64**	1,415,600	539,000	38.1%	2,076,100	686,500	33.1%

Source: Annual Population Survey (ONS 2021)

Disability

The UK prevalence of disability cases entitled to Personal Independence Payment (PIP) was sourced from

Table 5 – Personal Independence Payment entitlement from causes other than migraine

Age categories	Cases with PIP entitlement from non-migraine cause [a]		Total UK population [b]		Prevalence of PIP entitlement from non-migraine causes [c]	
	**Males**	**Females**	**Males**	**Females**	**Males**	**Females**
16 to 19	69,104	38,558	1,564,569	1,480,930	4.4%	2.6%
20 to 24	86,759	59,546	2,045,254	1,930,059	4.2%	3.1%
25 to 29	75,100	66,955	2,253,964	2,143,264	3.3%	3.1%
30 to 34	76,904	83,002	2,326,461	2,291,137	3.3%	3.6%
35 to 39	79,309	94,337	2,206,422	2,242,807	3.6%	4.2%
40 to 44	85,451	106,162	2,121,914	2,168,142	4.0%	4.9%
45 to 49	104,569	136,627	2,012,961	2,053,290	5.2%	6.7%
50 to 54	137,133	186,678	2,242,093	2,318,458	6.1%	8.1%
55 to 59	161,724	218,539	2,263,517	2,350,748	7.1%	9.3%
60 to 64	173,213	222,420	2,001,203	2,089,057	8.7%	10.6%
65 to 69	147,473	181,478	1,676,961	1,782,187	8.8%	10.2%
70 to 74	66,594	85,101	1,544,875	1,695,738	4.3%	5.0%
75 to 79	0	0	1,290,747	1,472,559	0.0%	0.0%
80 to 84	0	0	776,973	979,072	0.0%	0.0%
85 to 89	0	0	450,061	652,723	0.0%	0.0%
90+	0	0	215,265	422,547	0.0%	0.0%

[a] PIP cases with entitlement (ONS 2021).

[b] UK population (ONS 2022).

[c] Calculated by dividing cases with PIP entitlement from non-migraine causes by the total UK population for same age group.

2. Targeted literature search

The targeted literature search was conducted on PubMed in April 2022 using the migraine mesh heading and terms specific to labour market participation (above). The search was restricted to English language, European publications, and the last 10 years. Resulting titles and abstracts were sifted by a single analyst. Full texts were also inspected by a single analyst and manually checked for additional references. Studies reporting on relative measures of the impact of migraine on labour participation compared to the general population or less severe migraine were extracted and used in the model.

<u>PubMed literature search strategy</u>

#	Search terms	Hits
**1**	((((((("career mobility"[MeSH Terms] OR "caregiver burden"[MeSH Terms] OR "cost of illness"[MeSH Terms] OR "family leave"[MeSH Terms] OR "financial stress"[MeSH Terms] OR "retirement"[MeSH Terms] OR "return to work"[MeSH Terms] OR "social security"[MeSH Terms] OR "social welfare"[MeSH Terms] OR unemployment[MeSH Terms]) OR ("Activity impairment"[Title/Abstract] OR "Benefit payment"[Title/Abstract] OR Compensation[Title/Abstract] OR "Cost of illness"[Title/Abstract] OR "Disability allowance*"[Title/Abstract] OR Earning*[Title/Abstract] OR Fiscal[Title/Abstract] OR "Government transfer"[Title/Abstract] OR "Hidden cost*"[Title/Abstract] OR Income[Title/Abstract] OR "Indirect cost*"[Title/Abstract] OR "Indirect healthcare cost*"[Title/Abstract] OR "Living allowance*"[Title/Abstract] OR "Long-term disability"[Title/Abstract] OR "Lost time"[Title/Abstract] OR "Multifactor productivity"[Title/Abstract] OR Pension*[Title/Abstract] OR "Productive efficiency"[Title/Abstract] OR Retire[Title/Abstract] OR Retirement[Title/Abstract] OR Salaries[Title/Abstract] OR Salary[Title/Abstract] OR "Social benefit*"[Title/Abstract] OR "social insurance benefit"[Title/Abstract] OR "Social security"[Title/Abstract] OR "Tax credit"[Title/Abstract] OR "Total factor productivity"[Title/Abstract] OR "Transfer payment"[Title/Abstract] OR Transportation[Title/Abstract] OR Underemployment[Title/Abstract] OR "Under-employment"[Title/Abstract] OR Unemployment[Title/Abstract] OR "Un-employment"[Title/Abstract] OR Wage[Title/Abstract] OR Wages[Title/Abstract] OR Welfare[Title/Abstract] OR WPAI[Title/Abstract] OR "work life"[Title/Abstract] OR "workers compensation"[Title/Abstract] OR "workers comp"[Title/Abstract])) OR (((work*[Title/Abstract] OR job[Title/Abstract] OR occupation*[Title/Abstract] OR vocation*[Title/Abstract] OR employ*[Title/Abstract] OR workforce[Title/Abstract] OR "work-force"[Title/Abstract] OR "labor force"[Title/Abstract] OR "labour force"[Title/Abstract])) AND (Loss[Title/Abstract] OR productivity[Title/Abstract] OR disability[Title/Abstract] OR participation[Title/Abstract] OR cessation[Title/Abstract] OR status[Title/Abstract] OR outcome[Title/Abstract] OR impair*[Title/Abstract] OR disrupt*[Title/Abstract] OR incapacity[Title/Abstract] OR incapability[Title/Abstract] OR activity[Title/Abstract] OR transportation[Title/Abstract] OR leaving[Title/Abstract]))) OR ((caregiver) AND (work*[Title/Abstract] OR job[Title/Abstract] OR occupation*[Title/Abstract] OR vocation*[Title/Abstract] OR employ*[Title/Abstract] OR workforce[Title/Abstract] OR "work-force"[Title/Abstract] OR "labor force"[Title/Abstract] OR "labour force"[Title/Abstract]))) OR ((fewer[Title/Abstract] OR loss[Title/Abstract] OR lost[Title/Abstract] OR unpaid[Title/Abstract]) AND (work[Title/Abstract]) AND (day*[Title/Abstract] OR hour*[Title/Abstract]))))	323,363
**2**	("migraine disorders"[MeSH Terms]))	
**3**	#1 AND #2	423
**4**	("comparative effectiveness"[Title/Abstract] OR "drug cost*"[Title/Abstract] OR "treatment cost*"[Title/Abstract] OR "hospital cost*"[Title/Abstract] OR "health services cost*"[Title/Abstract] OR "medical service utilization"[Title/Abstract] OR "effectiveness analysis"[Title/Abstract] OR "case study"[Title/Abstract] OR "cost effectiveness"[Title/Abstract] OR "cost-effectiveness"[Title/Abstract]))	
**5**	#3 NOT #4	
**6**	("english"[Language])	
**7**	(("2011/12/01"[Date - Publication] : "3000"[Date - Publication])))	
**8**	#5 AND #6 AND #7	
**9**	“Europe”[MeSH] OR “United Kingdom”[tiab] OR “UK” OR “Engl*”[tiab] OR “British”[tiab] OR “London” OR “Europe*” [tiab]	
**10**	#8 AND #9	95

3. Occupational burden of migraine and model implementation

Employment

People with migraine were subject to the effect of the condition on labour participation. The likelihood of employment was reduced from baseline values by 7% in people with episodic migraine and by 19% in people with chronic migraine (Stewart, Wood et al. 2010). These individuals were assumed to be in unemployment. The proportion of part-time workers due to chronic migraine was increased by 28% (Buse, Manack et al. 2010). Reductions of 19.7% or 12.6% in monthly working days (full- or part-time) were linked to chronic or episodic migraine, respectively. These were applied to the earnings of public sector employees and self-employed workers (Vo, Fang et al. 2018).

Absenteeism

Absenteeism metrics reported by Vo and colleagues (Vo, Fang et al. 2018) were applied to people affected by migraine on employment. In the cohort unaffected by migraine, a pre-COVID19 (year 2019) absenteeism rate of 1.9% was used. (ONS 2023) We assumed that employees in the public or private sector had their salaries protected despite episodes of absenteeism. The monetary value of absenteeism in public employees was assumed to represent a direct loss to the government and was included as a negative monetary value in the results. In people who were self-employed, absenteeism was assumed to represent a real reduction in earnings indirectly impacting the government through reduced direct and indirect tax contributions.

Early retirement

People with chronic migraine were modelled to have 1.611 higher probability of early retirement, compared to people with episodic migraine (Chalmer, Hansen et al. 2020).

Disability

The proportion of migraine-related disability was informed by data from the Department of Work and Pensions (ONS 2021). The proportion of individuals entitled to Personal Independence Payment (PIP) due to migraine were calculated by dividing the count of cases with entitlement by the total UK migraine population (Table 6). We assumed that there would be no migraine related disability in people not affected by migraine but that people with migraine would still be subject to disability from other causes.

People with chronic migraine were modelled to have 1.8 times higher probability of disability than people with episodic migraine (Buse, Manack et al. 2010).

Table 6 – Personal Independence Payment, cases with entitlement due to migraine

**Age categories**	**Cases with PIP entitlement [a]**		**Total UK migraine population by age [b]**		**Proportion of migraine population receiving PIP [c]**	
	**Males**	**Females**	**Males**	**Females**	**Males**	**Females**
16 to 19	14	31	244,073	515,364	0.006%	0.006%
20 to 24	24	72	319,060	671,661	0.008%	0.011%
25 to 29	40	141	351,618	745,856	0.011%	0.019%
30 to 34	58	215	362,928	797,316	0.016%	0.027%
35 to 39	61	271	344,202	780,497	0.018%	0.035%
40 to 44	85	297	331,019	754,513	0.026%	0.039%
45 to 49	81	348	314,022	714,545	0.026%	0.049%
50 to 54	99	347	260,083	544,838	0.038%	0.064%
55 to 59	75	285	262,568	552,426	0.029%	0.052%
60 to 64	71	207	232,140	490,928	0.031%	0.042%
65 to 69	37	79	194,527	418,814	0.019%	0.019%
70 to 74	14	22	95,782	147,529	0.015%	0.015%
75 to 79	0	0	80,026	128,113	0	0
80 to 84	0	0	48,172	85,179	0	0
85 to 89	0	0	27,904	56,787	0	0
90+	0	0	13,346	36,762	0	0

[a] PIP cases with entitlement (ONS 2021).

[b] Calculated by multiplying the age and gender-specific prevalence of migraine (The Work Foundation 2018) by the number of individuals for that age category in the UK population (ONS 2022).

[c] *Calculated by dividing cases with PIP entitlement by the total UK migraine population for same age group.*

4. Fiscal consequences inputs

Earnings from employment

The UK median monetary values of earnings from employment before tax are shown in Table 7.

Table 7 – Median gross earnings in the UK

Age categories	Full time		Part time	
	**Males**	**Females**	**Males**	**Females**
**16 to 17**	£9,062	£7,745	£3,210	£3,101
**18 to 21**	£18,392	£17,005	£6,780	£5,912
**22 to 29**	£26,856	£25,115	£10,561	£10,153
**30 to 39**	£34,210	£30,540	£12,500	£12,151
**40 to 49**	£38,463	£31,679	£12,000	£12,479
**50 to 59**	£36,000	£28,811	£11,773	£12,018
**60+**	£30,944	£24,850	£11,863	£10,547

Source: House of Commons Library (Francis-Devine 2021)

Absenteeism costs

To calculate absenteeism costs, the distribution of public and private employees, and self-employed workforce was considered (Table 1,Table 2,Table 3). The inputs used to define the distribution and sex of direct care providers in the health and social care sector can be found in Table 8.

Table 8 – Inputs used to model absenteeism

Description	Input	Source
**Public sector workforce**		
Public sector workforce headcount	5,674,000	(ONS 2021)
NHS workforce headcount	1,846,000	(ONS 2021)
SC workforce headcount	208,000	(Skills for Care 2022)
**Gender distribution NHS and SC**		
NHS - Males	325,415	(ONS 2021)
SC - Males	193,800	(Skills for Care 2022)
**Direct care providers**		
NHS workforce	719,291	(NHS Digital 2022)
Social care workforce (publicly and privately funded)	59.97%	Calculated [a]
**Gender distribution of direct care providers**		
Direct carers, NHS, Males	167,155	(NHS Digital 2022)
Direct carers, SC, Males (publicly and privately funded)	17.00%	[b]

Acronyms: NHS, National Health System; SC, Social Care

[a] Headcount for publicly funded SC direct care providers was not found in the literature. Combined input for publicly and privately funded SC used instead (134,400/224,100) (Skills for Care 2022).

[b] Headcount for male direct care providers in publicly funded SC was not found in the literature. Combined input for publicly and privately funded SC used instead (Skills for Care 2022).

Personal Independence Payment (PIP)

People with disability in the population affected by and unaffected by migraine were assigned the weekly value of personal independence payment (PIP) from all disability causes. People with migraine-related excess disability in the migraine cohort were assigned the weekly value of PIP due to migraine alone (ONS 2021).

Table 9 – Weekly monetary value of personal independence payment

Age categories	Migraine		All other causes of disability	
	**Males**	**Females**	**Males**	**Females**
16 to 19	£124.18	£104.59	£110.92	£107.75
20 to 24	£97.55	£95.71	£127.92	£124.75
25 to 29	£99.96	£100.44	£118.50	£112.58

30 to 34	£96.62	£100.58	£112.17	£106.08
35 to 39	£105.59	£98.26	£108.08	£103.42
40 to 44	£110.22	£98.69	£106.08	£103.17
45 to 49	£103.76	£100.86	£105.17	£103.75
50 to 54	£109.30	£101.31	£106.75	£105.42
55 to 59	£107.73	£106.15	£108.17	£106.92
60 to 64	£105.68	£108.62	£109.75	£108.17
65 to 69	£105.31	£96.75	£110.83	£109.08
70 to 74	£129.87	£106.98	£111.00	£109.75
75 to 79	£0.00	£0.00	£113.00	£112.00
80 to 84	£0.00	£0.00	£0.00	£0.00
85 to 89	£0.00	£0.00	£0.00	£0.00
90+	£0.00	£0.00	£0.00	£0.00

Source: Department of Work and Pensions (ONS 2021)

Pensions

Normal retirement pensions were thought not to differ between people affected and unaffected by migraine and were not included in the model. Individuals retiring early were assumed to receive state pension transfers (ONS 2021). The weekly age-adjusted monetary value for state pension is shown in Table 10.

Table 10 – Average monetary value of state pension age

Age categories	Weekly pension
65-69	£158.90
70-74	£150.63
75-79	£150.15
80-84	£157.60
85-89	£164.29
90 and over	£157.50

Source: Office for National Statistics (ONS 2021)

Direct healthcare costs

The average cost of managing one or more episodes of migraine was sourced from the NICE single technology appraisal for erenumab (NICE 2021). An average annual direct healthcare cost was calculated using the cost value per health state column (Table 11) weighted by the distribution of monthly migraine days (MMDs) shown in Table 13. An annual direct healthcare cost of £897 was obtained for use in the model. In the absence of UK specific data, the proportion of individuals using healthcare resources paid by the government (46%) was sourced from international studies (Lipton, Serrano et al. 2012, Groth, Katsarava et al. 2022).

Table 11 – Annual healthcare resource use and cost by frequency of monthly migraine days [a]

Monthly migraine days	General practitioner visits	Emergency department visits	Hospitalizations	Nurse practitioner visits	Neurologist visits	Oral triptan usage	Cost value per health state [b]
0	2.622	0.390	0.303	0.823	0.043	0.000	£388.88
1	3.748	0.867	0.542	1.322	0.195	3.839	£699.16
2	3.748	0.867	0.542	1.322	0.195	10.261	£704.00
3	3.748	0.867	0.542	1.322	0.195	16.683	£708.83
4	5.373	0.758	0.520	2.275	0.173	23.105	£785.41
5	5.373	0.758	0.520	2.275	0.173	29.527	£790.24
6	5.373	0.758	0.520	2.275	0.173	35.949	£795.07
7	5.373	0.758	0.520	2.275	0.173	42.371	£799.90
8	7.193	1.192	0.520	0.628	0.498	48.793	£924.89
9	7.193	1.192	0.520	0.628	0.498	55.215	£929.73
10	7.193	1.192	0.520	0.628	0.498	61.637	£934.56
11	7.193	1.192	0.520	0.628	0.498	68.059	£939.39
12	7.193	1.192	0.520	0.628	0.498	74.481	£944.22
13	7.193	1.192	0.520	0.628	0.498	80.903	£949.05
14	7.193	1.192	0.520	0.628	0.498	87.325	£953.88

15	7.605	1.517	0.672	1.647	0.953	93.747	£1,245.90
16	7.605	1.517	0.672	1.647	0.953	100.169	£1,250.73
17	7.605	1.517	0.672	1.647	0.953	106.591	£1,255.56
18	7.605	1.517	0.672	1.647	0.953	113.013	£1,260.39
19	7.605	1.517	0.672	1.647	0.953	119.435	£1,265.23
20	7.605	1.517	0.672	1.647	0.953	125.857	£1,270.06
21	7.605	1.517	0.672	1.647	0.953	132.279	£1,274.89
22	7.605	1.517	0.672	1.647	0.953	138.701	£1,279.72
23	7.605	1.517	0.672	1.647	0.953	145.123	£1,284.55
24	7.605	1.517	0.672	1.647	0.953	151.545	£1,289.38
25	7.605	1.517	0.672	1.647	0.953	157.967	£1,294.22
26	7.605	1.517	0.672	1.647	0.953	164.385	£1,299.04
27	7.605	1.517	0.672	1.647	0.953	170.807	£1,303.88
28	7.605	1.517	0.672	1.647	0.953	177.229	£1,308.71

[1] Reproduced from Committee Papers for NICE Single Technology Appraisal - erenumab for preventing migraine [ID1188]. Shown values are for an annual cycle.

[2] Calculated using row proportions and unit costs from Table 12.

Table 12 – Unit costs of healthcare resources

Resource	Unit cost	Source	Notes
General practitioner visit	£39.00	PSSRU 2021	Per surgery consultation lasting 9.22 minutes
Emergency department visit	£123.84	NHS Reference costs	[VB09Z] Emergency Medicine, Category 1 Investigation with Category 1-2 Treatment
Hospitalization	£643.29	NHS Reference costs	Weighted average of Total HRG [AA31C], [AA31D] and [AA31E]
Nurse visit	£42.00	PSSRU 2021	One hour of nurse time in a general practitioner surgery
Neurologist visit	£199.00	NHS Reference costs	Outpatient attendance [400] Neurology - Unit cost - Consultant led
Triptan use	£0.75	Prescription Costs Analysis 2022, NHS Drug Tariff 2022	Average cost for one triptan tablet

Acronyms: HRG, healthcare resource groups; NHS, National Health System; PSSRU, Personal Social Services Research Unit.

5. Monthly migraine days

The base case ratio of chronic to EM cases was sourced from the UK cohort in the International Burden of Migraine Study (IBMS) (Bloudek, Stokes et al. 2012) as this was perceived to be more representative of the overall migraine population in the UK. Nonetheless, the distribution of MMDs was also required to calculate healthcare costs and to model scenarios exploring the hypothetical effect of therapies reducing the frequency of MMDs, which was not reported in the publication by Bloudek and colleagues.

Alternatively, histograms published by Di Tanna et al. (Di Tanna, Porter et al. 2019) were digitized to inform the proportion of individuals experiencing migraine over a month. The digitized data refers to for individuals assigned to Placebo, classified to have EM or CM at week 0, figures 1 and 2 of the original publication.

The digitized inputs and manipulations produced to the data are shown in Table 13.

Table 13 – Distribution of Monthly migraine days

	MMDs	Di Tanna 2019 [a]	Reweighted [b]	Distribution of MMDs [c]
**EM**	0	0.00%	0.00%	0.00%
	1	0.00%	0.00%	0.00%
	**2**	0.00%	0.00%	0.00%
	**3**	0.00%	0.00%	0.00%
	**4**	3.33%	3.35%	3.17%
	**5**	8.92%	8.95%	8.48%
	**6**	18.76%	18.83%	17.83%
	**7**	10.65%	10.69%	10.12%
	**8**	14.25%	14.30%	13.54%
	**9**	13.58%	13.63%	12.91%
	**10**	10.86%	10.90%	10.32%
	**11**	7.81%	7.83%	7.42%
	**12**	7.48%	7.50%	7.10%
	**13**	1.23%	1.23%	1.17%
	**14**	2.76%	2.77%	2.62%
**CM**	**15**	6.09%	7.96%	0.42%
	**16**	5.72%	7.48%	0.40%
	**17**	8.53%	11.14%	0.59%
	**18**	9.68%	12.65%	0.67%
	**19**	7.51%	9.81%	0.52%
	**20**	8.94%	11.69%	0.62%
	**21**	3.56%	4.65%	0.25%
	**22**	5.06%	6.61%	0.35%
	**23**	7.52%	9.83%	0.52%
	**24**	3.57%	4.66%	0.25%
	**25**	2.89%	3.77%	0.20%
	**26**	2.11%	2.76%	0.15%
	**27**	2.89%	3.78%	0.20%
	**28**	2.46%	3.21%	0.17%

[a] Digitized using WebPlotDigitizer (Rohatgi 2021).

[b] Row proportions were reweighted to add to 100% of EM or to 100% of CM (i.e. 3.33%/[3.33%+8.92%+18.76%+10.65%+14.25%+13.58%+10.86%+7.81%+7.48%+1.23%+2.76%]).

[c] Reweighted values for EM were multiplied by the proportion of individuals with EM (94.7%) sourced from the IBMS (Payne, Varon et al. 2011). Similarly, reweighted values for CM were multiplied by 5.3%.

6. Additional results

The population model used published prevalence metrics (The Work Foundation 2018) of migraine and the total UK population (ONS 2022) to predict the total number of males and females with the condition in one single year. The resulting cohort is shown in Figure 1.

Figure 1 – Predicted prevalence of migraine in the UK by age and sex

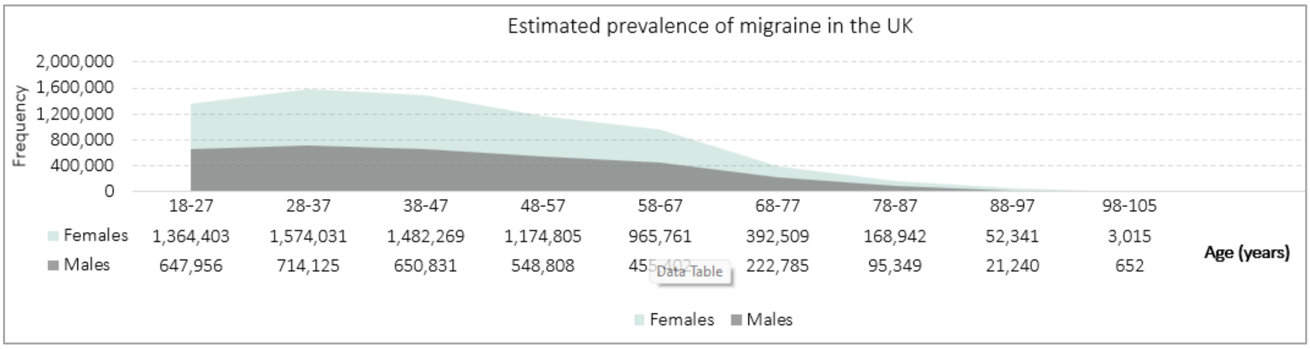

The population model estimated the incremental fiscal consequences due to migraine in the cohort depicted in Figure 1, compared to an identical cohort unaffected by the disease. The disaggregated base case results are reported in Table 14.

Table 14 – Population model base case results

		Migraine population	General population	Incremental		
	Gross income from any employment	£173,921 M	£179,771 M	-£5,850 M		
**Fiscal consequences**	Public sector absenteeism	-£4,211 M	-£631 M	-£3,580 M	29.3%	Fiscal loss
	Direct taxes from employment	£52,872 M	£54,650 M	-£1,778 M	14.6%	Fiscal loss
	Indirect taxes from employment	£21,566 M	£22,292 M	-£725 M	5.9%	Fiscal loss
	Foregone corporation taxes	-£1,515 M	-£227 M	-£1,288 M	10.6%	Fiscal loss
	Job seeker's allowance	-£1,630 M	-£1,173 M	-£457 M	3.7%	Fiscal loss
	Early retirement pension	-£601 M	-£582 M	-£19 M	0.2%	Fiscal loss
	Disability pension	-£3,140 M	-£3,128 M	-£12 M	0.1%	Fiscal loss
	Indirect tax from transfers	£1,629 M	£1,625 M	£4 M	0.0%	Fiscal gain
	Healthcare costs	-£24,203 M	-£19,857 M	-£4,346 M	35.6%	Fiscal loss
	**Total**	£40,766 M	£52,968 M	-£12,202 M		
	Life years	10,535,224	10,535,224	0.000		
	Incremental costs per life year lived with migraine			**-£1,158**		

Acronyms: IFC, Incremental fiscal consequences

Negative values represent monetary losses and positive values sources of revenue to the UK government.

Table 15 – Population model estimates of UK fiscal costs of absenteeism

					Fiscal costs of absenteeism			
**Sectors of UK workforce**			**Head-count**	**% UK workforce**	**Migraine population**	**General population**	**Incremen-tal**	**%**
Public sector workforce	Health and Social Care	Replacement costs (Direct carers)	2,054,000	6.1%	-£855 M	-£128 M	-£727 M	12.5%
		Foregone labour value (Not direct carers)			-£1,560 M	-£234 M	-£1,326 M	22.8%
	Other	Foregone labour value	3,620,000	10.8%	-£1,797 M	-£269 M	-£1,527 M	26.3%
Private sector self employed		Direct tax on employment	3,677,550	11.0%	-£787 M	-£118 M	-£668 M	11.5%
		Indirect tax on employment			-£321 M	-£48 M	-£273 M	4.7%
Private sector employees		Foregone corporate tax contributions	24,230,750	72.2%	-£1,515 M	-£227 M	-£1,288 M	22.2%
**Total absenteeism costs**			**33,582,300**	**100.0%**	**-£6,835 M**	**-£1,025 M**	**-£5,810 M**	**100.0%**

7. References

Bloudek, L. M., M. Stokes, D. C. Buse, T. K. Wilcox, R. B. Lipton, P. J. Goadsby, S. F. Varon, A. M. Blumenfeld, Z. Katsarava, J. Pascual, M. Lanteri-Minet, P. Cortelli and P. Martelletti (2012). "Cost of healthcare for patients with migraine in five European countries: results from the International Burden of Migraine Study (IBMS)." J Headache Pain **13**(5): 361-378.

Buse, D. C., A. Manack, D. Serrano, C. Turkel and R. B. Lipton (2010). "Sociodemographic and comorbidity profiles of chronic migraine and episodic migraine sufferers." J Neurol Neurosurg Psychiatry **81**(4): 428-432.

Chalmer, M. A., T. F. Hansen, E. R. Lebedeva, D. W. Dodick, R. B. Lipton and J. Olesen (2020). "Proposed new diagnostic criteria for chronic migraine." Cephalalgia **40**(4): 399-406.

Di Tanna, G. L., J. K. Porter, R. B. Lipton, A. Brennan, S. Palmer, A. J. Hatswell, S. Sapra and G. Villa (2019). "Migraine day frequency in migraine prevention: longitudinal modelling approaches." BMC Med Res Methodol **19**(1): 20.

Francis-Devine, B. (2021). "Research briefing - Average earnings by age and region." Retrieved 10/05/2022, from https://commonslibrary.parliament.uk/research-briefings/cbp-8456/.

Groth, M., Z. Katsarava and M. Ehrlich (2022). "Results of the gErman migraine PatIent Survey on medical Care and prOPhylactic treatment Experience (EPISCOPE)." Scientific Reports **12**(1): 4589.

Lipton, R. B., D. Serrano, S. Holland, K. M. Fanning, M. L. Reed and D. C. Buse (2012). "Barriers to the diagnosis and treatment of migraine: effects of sex, income, and headache features." Headache **53**(1): 81-92.

NHS Digital (2022). "NHS Hospital & Community Health Service (HCHS) monthly workforce statistics - Staff in NHS Trusts and other core organisations."

NICE. (2021). "Single Technology Appraisal Erenumab for preventing migraine [ID1188] Committee Papers [TA 682]." Retrieved 18/12/2022, from https://www.nice.org.uk/guidance/ta682/evidence/appraisal-consultation-committee-papers-pdf-9021642589.

ONS. (2018). "Age distribution of UK civil servants " Retrieved 21/04/2022, from https://www.nomisweb.co.uk/datasets/acses4i

ONS. (2021). "Annual Population Survey - Regional - Economic inactivity by reasons." Retrieved 10/05/2022, from https://www.nomisweb.co.uk/

ONS. (2021). "Annual Population Survey - regional - labour market status by age." Retrieved 20/04/2022, from https://www.nomisweb.co.uk/datasets/aps170

ONS. (2021). "Pensions by age (bands and single year) by Quarter by Mean of Weekly Amount of Benefit." Retrieved 23/05/2022, from https://stat-xplore.dwp.gov.uk/webapi/jsf/tableView/tableView.xhtml

ONS. (2021). "Personal Independence Payment - Cases with Entitlement by age categories." Retrieved 04/05/2022, from https://stat-xplore.dwp.gov.uk/webapi/jsf/login.xhtml.

ONS. (2021). "Personal Independence Payment - Mean of financial award for people with migraine." Retrieved 04/05/2022, from https://stat-xplore.dwp.gov.uk/webapi/jsf/login.xhtml.

ONS. (2021). "UK Public sector employment by industry." Retrieved 21/04/2022, from https://www.ons.gov.uk/employmentandlabourmarket/peopleinwork/publicsectorpersonnel/datasets/publicsectoremploymentreferencetable

ONS. (2022). "National Population projections by single year of age." Retrieved 11/05/2022, from https://www.nomisweb.co.uk/datasets/ppsyoa.

ONS. (2023). "Sickness absence in the UK labour market." Retrieved 10/06/2023, from https://www.ons.gov.uk/employmentandlabourmarket/peopleinwork/employmentandemployeetypes/datasets/sicknessabsenceinthelabourmarket.

Payne, K. A., S. F. Varon, A. K. Kawata, K. Yeomans, T. K. Wilcox, A. Manack, D. C. Buse, R. B. Lipton, P. J. Goadsby and A. M. Blumenfeld (2011). "The International Burden of Migraine Study (IBMS): Study design, methodology, and baseline cohort characteristics." Cephalalgia **31**(10): 1116-1130.

Rohatgi, A. (2021). "WebPlotDigitizer 4.5." Retrieved 19/08/2022, from https://automeris.io/WebPlotDigitizer.

Skills for Care. (2022). "Adult social care workforce estimates, Employment overview." Retrieved 04/11/2022, from https://www.skillsforcare.org.uk/Adult-Social-Care-Workforce-Data/Workforce-intelligence/publications/national-information/The-state-of-the-adult-social-care-sector-and-workforce-in-England.aspx.
Stewart, W. F., G. C. Wood, A. Manack, S. F. Varon, D. C. Buse and R. B. Lipton (2010). "Employment and work impact of chronic migraine and episodic migraine." J Occup Environ Med **52**(1): 8-14.
The Work Foundation. (2018). "Society's headache: The socioeconomic impact of migraine " Retrieved 26/04/2022, from https://www.lancaster.ac.uk/media/lancaster-university/content-assets/documents/lums/work-foundation/SocietysHeadacheTheSocioeconomicimpactofmigraine.pdf.
Vo, P., J. Fang, A. Bilitou, A. K. Laflamme and S. Gupta (2018). "Patients' perspective on the burden of migraine in Europe: a cross-sectional analysis of survey data in France, Germany, Italy, Spain, and the United Kingdom." J Headache Pain **19**(1): 82.
